# The use of eponyms in medical case reports: etymological, quantitative, and structural analysis

**DOI:** 10.1186/s13256-023-03895-0

**Published:** 2023-04-06

**Authors:** Yuliia Lysanets, Olena Bieliaieva

**Affiliations:** Department of Foreign Languages with Latin and Medical Terminology, Poltava State Medical University, Poltava, Ukraine

**Keywords:** Medical discourse, Medical case report, Eponyms, Term, Greek and Roman mythologies

## Abstract

**Background:**

The present paper focuses on eponyms, that is, terms with proper names, in particular, derived from world mythologies, the Bible, and modern literature. The study highlights the significance of this terminological phenomenon in the English sublanguage of medicine and discusses its role in the process of writing medical case reports. The objectives of the research are to study the prevalence of eponyms in the English language in medical case reports and to analyze the etymology of the revealed terms. The deeper purpose of our study is to demonstrate that eponymic terms in general, and mythological and literary eponyms, in particular, are present in doctors’ spoken and written discourse far more extensively than might seem at first glance. By drawing attention to this terminological phenomenon, we will provide relevant guidelines, which will ensure the correct use of eponyms by medical professionals who will deal with the genre of medical case reports.

**Methods:**

We studied the prevalence of these terms in the issues of *Journal of Medical Case Reports* (2008–2022) and classified them according to their etymological origin and frequency of use. The selected medical case reports were considered using the methods of quantitative examination, and structural, etymological, and contextual analyses.

**Results:**

We detected the major tendencies in using mythological and literary eponyms in medical case reports. We found a total of 81 mythological and literary eponyms, represented by 3995 cases of use in *Journal of Medical Case Reports* issues, and traced the etymology of their onomastic components. Hence, we delineated the five most prevalent sources of these terminological units: Greek mythology, Roman mythology, other world mythologies, the Bible, and fiction. The research revealed that modern medical case reports largely rely primarily on Greek mythology (65 eponyms, 3633 results), which is due to a rich informational and metaphorical arsenal of these ancient corpora of human knowledge. The group of eponyms rooted in Roman mythology ranks second, and these terms are much less prevalent in modern medical case reports (6 eponyms, 113 results). Four eponyms (88 results) represent other world mythologies (Germanic and Egyptian). Two terms with onomastic components come from the Bible (15 results), and four eponyms stem from modern literature (146 results). We also detected several widespread mistakes in the spelling of some mythological and literary eponyms. It is our opinion that the awareness of an eponym’s etymology can effectively prevent and minimize the appearance of such errors in medical case reports.

**Conclusions:**

The adequate use of mythological and literary eponyms in medical case reports is an effective way to share one’s clinical findings with colleagues from all over the world, because these eponyms are internationally widespread and understood. Correct use of eponyms promotes the continuity of medical knowledge and ensures conciseness and brevity, which are indispensable features of medical case reports as a genre. Therefore, it is highly important to draw students’ attention to the most prevalent mythological and literary eponyms, used in contemporary medical case reports, so they could use them appropriately, as well as with due awareness of the origin of these terms. The study also demonstrated that medicine and humanities are closely related and inherently interconnected areas. We believe that the study of this group of eponyms should be an integral component of doctors’ training and continuing professional education. This will ensure the interdisciplinary and synergic approach in modern medical education, which in turn will promote the all-round development of future healthcare specialists, endowed not only with professional expertise, but also with extensive background knowledge.

## Background

Eponym [Ancient Greek ἐπώνυμος (epṓnymos), from ἐπί (epí, “upon”) + ὄνυμα (ónyma, “name,” also referred to as “term with onomastic component”: ὀνομαστικός (onomastikós) —“belonging to naming”] is a word derived from the name of a person (real or fictitious) [1]. In particular, medical eponyms may denote diseases, structures, operations, or procedures. Medical eponyms constitute “an integral part of the language of medicine, which reflects the various stages of knowledge of reality, historical conditions, and information about those people who contributed to the development of medical science” [2]. The significance of eponyms derived from the names of doctors and scientists who discovered or described them first has been extensively studied in different medical specialties [3–7]. There are studies devoted to ethical [8–10], historical [2, 11], and gender [12] aspects of medical eponyms. Along with linguistic studies, eponyms are the constant focus of medical research. Indeed, professional medical discourse actively uses eponyms, which are derived from the names of prominent scientists and doctors, as is the case with “Heymann nephritis” [13], “Parkinson’s disease” [14], “Devic’s opticomyelitis” [15], “Adams–Stokes disease” [16], “Aschner–Dagnini reflex” [17], and many others. At the same time, in the context of our research, it is essential to point out that along with such “obvious” derivations, there are numerous terms with onomastic components, whose etymology might not be so transparent at first glance. For instance, the term “*Bordetella avium*” is derived from the name of the Belgian immunologist and microbiologist Jules Bordet (1870–1961), and “*Raoultella planticola*” is named after the French bacteriologist Didier Raoult (born 1952) [18]. The term “warfarin” is derived from the abbreviation “Wisconsin Alumni Research Foundation” (WARF), which is a homage to the team of chemists from the University of Wisconsin [19]. Furthermore, the term “fullerene” is derived from the name of American architect Buckminster Fuller, because of the similarity of its molecule to geodesic domes, which he designed [20]. As one can observe, the above-mentioned onomastic components require certain interpretative effort and address reference literature. Quite often, unawareness of a term’s intricate etymology can result in its incorrect spelling and use. For instance, our searches in the ResearchGate and PubMed databases yield more than ten results for each of the misspelled terms “fulerene”, “*Bortedella*”, and “*raultella*”. It is our belief that the knowledge of the etymological roots of such terms can help healthcare professionals to avoid possible mistakes when producing written professional discourse. Therefore, medical students must be appropriately instructed as to these tendencies in English medical terminology as a part of their formal education.

Eponyms are widely used in medical discourse because they “disclose the evolution of medical research and practice; provide continuity of scientific knowledge and contribute to the formation of terminological competence of medical students” [21]. Indeed, eponyms “bring colour to medicine, embed medical traditions and culture to our history” [22]. However, the features and prevalence of eponyms embedded in classical mythologies and world literature remain understudied, so far. At the same time, the paramount role of the Greek and Latin terminologies in the development of all modern European languages, and their enormous and undisputable influence on the English sublanguage of medicine, have been thoroughly substantiated in our previous studies [23–28]. In addition, several studies have focused on the origins of specific literary eponyms [29], or their prevalence in certain medical specialties [30, 31]. Therefore, our study aims to fill this research gap and deepen doctors’ and linguists’ understanding of this terminological phenomenon, hence the interdisciplinary nature of this research. In our opinion, the study of eponyms derived from the cultural background of different peoples and nations is an indispensable element of the well-rounded education of medical specialists in general [32], and an essential prerequisite for further integration of Ukraine into the Western context. It is also a feasible tool for increasing students’ motivation, since the study of eponyms contribute to the understanding of medical terms’ appearance, and consequently promotes their better memorization [33].

Within the structure of medical writing, a special place belongs to medical case reports (MCRs), which are highly effective in the communication of scientific knowledge throughout the world, hence the importance of training medical professionals in producing written discourse in this genre [34]. Therefore, it is relevant to determine the prevalence of eponymic terms and how they are used in the modern MCRs. In such a way, this research will provide appropriate guidelines for healthcare professionals in terms of correct use and comprehension of the origins of medical eponyms, when dealing will the genre of MCRs.

## Methods

The objective of the research is to study the prevalence of using mythological and literary eponyms in the English language medical case reports and, in such a way, to provide academic guidelines for writing effective MCRs by medical professionals. The deeper purpose of our study is to demonstrate that eponymic terms in general, mythological, and literary eponyms, in particular, are present in doctors’ spoken and written discourse far more extensively than might seem at first glance. The material of this research is the corpus of papers from *Journal of Medical Case Reports* (*JMCRs*) [35], published within the last 15 years (2007–2022). The choice and scope of the researched material are determined by the fact that the journal was established in 2007, and it is a leading open-access peer-reviewed platform, where the major tendencies within the genre of MCRs can be effectively traced and analyzed. Our particular interest in MCRs stems from the highly important role of this genre in medical communication and education. MCRs enable the rapid exchange of essential clinical data between researchers from all over the world. This ensures the advance of global science and healthcare. We selected mythological and literary eponyms in medicine, using The Latin-Ukrainian Thesaurus of Clinical Terms [36], The Latin-Ukrainian Medical Encyclopedic Dictionary [1], and Medicine, Literature and Eponyms: An Encyclopedia of Medical Eponyms Derived from Literary Characters [37]. Further, we checked the presence and prevalence of each mythological and literary eponym in *JMCRs* by automatic search on the journal’s website [section “Articles”—“Search articles within this journal”—“All volumes” (2007–2022)].

The selected medical case reports were considered using the methods of quantitative examination, structural, etymological, and contextual analyses. The method of quantitative examination involves counting elements in the researched material to determine numerical trends and patterns. In the context of medical case reports, this method allows for collecting data on the frequency of eponymic terminological units. The structural method aims to describe and analyze the constituent parts and properties of a word at various levels, for example, its morphology (word-building features), syntax (the rules governing the formation of phrases), and semantics (the study of the meaning of words, phrases, and sentences in language). Structural analysis is a powerful tool for understanding the underlying patterns and regularities of language and its role in human communication. Etymological analysis reveals the origins and evolution of words and language. In medical discourse, etymological analysis involves examining the history and usage of medical terminology, and using this knowledge to better understand the meaning and significance of certain terms and terminological collocations. Contextual analysis implies examining and deeper understanding of a term within its broader historical, social, and cultural setting. For eponyms, this might involve considering factors, such as the time period in which the terms were coined for the first time, the cultural or societal beliefs that may have influenced medical practices at the time, or the broader medical context in which a term was used.

To analyze eponymic concepts quantitatively, we designed an etymological typology. We identified the most common etymological sources and recorded the number of results each detected eponym was used in *JMCRs*. Hence, we delineated the five most prevalent sources of these terminological units: Greek mythology, Roman mythology, other world mythologies, the Bible, and fiction. For structural consideration of the material under study, we grouped the detected eponyms into three categories: (1) one-word terms, (2) two-word phrases, and (3) three-word phrases. We used this classification due to the specificity of word-building patterns in English medical terminology, in which eponyms are generally represented by these three categories [24]. Moreover, this grouping ensures clarity and structural coherence as it enabled us to compare and contrast different types of eponyms, identify any trends that emerge across the different categories, and detect structural patterns within the researched material. Further, we organized the selected material into five semantic groups and determined their frequency in *JMCRs*: (1) pathological conditions, (2) anatomical descriptions, (3) physiological conditions, (4) medical instruments, diagnostic tools, and doctor’s manipulations, and (5) chemical substances and medications. These five semantic groups cover virtually all spheres of the medical profession. On the one hand, this grouping facilitates students’ memorizing of eponymic terminological material in a well-structured and meaningful manner, rather than a continuous flow of information. This will be beneficial, not only for enhancing students’ terminological competence, but also for their mastering of various clinical and theoretical subjects in medicine, for example, anatomy, physiology, pathophysiology, therapy, surgery, pharmacology, and so on, hence the interdisciplinary value of this classification. On the other hand, this approach vividly reflects which spheres of medicine have been influenced by mythological and literary eponyms most and least of all, thus contributing to a deeper analysis of terminological features and tendencies in medical discourse. By grouping the selected material into these five categories, we have achieved organizational clarity and semantic coherence for analyzing the data. By breaking the material down into smaller, more manageable units, we could more easily identify key patterns within the data. Furthermore, by categorizing the selected material into five semantic groups, we were aiming to facilitate the practical application of findings in clinical or research settings. For example, we have observed that certain types of terms were more common in certain contexts or disciplines (such as pathological conditions), while others were more prevalent in others (such as anatomical descriptions). Hence, by identifying the most common types of pathological conditions or chemical substances in the eponymic material, the study can provide valuable insights for clinicians and researchers working in these areas.

## Results

We found a total of 81 mythological and literary eponyms, represented by 3995 cases of use in *JMCRs* issues, and traced the etymology of their onomastic components. The research revealed that modern medical case reports largely rely primarily on Greek mythology (65 eponyms, 3633 results). Table 1 presents our findings: the Greek eponym, along with the reference information about it, the number of results, and some examples of its use in *JMCRs* as of 2022, as well as noneponymous equivalents (if available in medical terminology), also found in *JMCRs*.

**Table 1:** Eponyms rooted in the Greek mythology

No.	Terms with onomastic components	Number of results in *JMCRs* as of 2022	Examples from *JMCRs*	Reference information	Alternative terms (if available)
1.	Achilles tendon	42 results	“We present an interesting case of spontaneous non-traumatic bilateral rupture of the *Achilles tendons*, which is a rare condition” [35]	**Achilles** is one of the main ancient Greek heroes. According to the myth, Achilles was the son of King Peleus and the sea goddess (Nereid) Thetis, who, seeking to make her son immortal, bathed him as a baby in the waters of the underground river Styx, holding his heel. It was the heel that became the vulnerable place in which the arrow of Paris hit during the Trojan War—this is where the expression “Achilles’ heel” comes from, that is, a weak, vulnerable place [36].	**Calcaneal tendon** (2 results of *JMCRs*)
2.	Achilles tendon reflex	9 results	“Her knee jerks were brisk, and her *Achilles tendon reflexes* were difficult to elicit” [35]		**Ankle-jerk reflex, ankle jerk reflex** (5 results in *JMCRs*)
3.	Arthralgia	154 results	“Three days prior to admission he developed high spiking fever with chills and rigors associated with severe *arthralgia* and *myalgia*” [35]	The Greek suffix ***-algia*** means “pain” and is derived from the name of **Algaea**—the daughter of Eris, goddess of war and strife, and Eiter, the god of misty and cloudy skies. Algaea personified pain, and suffering of the body and mind, which were intensified by grief [36]	** Joint pain** (629 results in *JMCRs*), **painful joints** (5 results)
4.	Myalgia	159 results			** Muscle ache** (10 results in *JMCRs*), **muscle pain** (40 results), **muscular pain** (5 results)
5.	Neuralgia	47 results	“The result with respect to his sporadic neuralgia was satisfactory” [35]		** = Nerve pain** (6 results in *JMCRs*)
6.	Fibromyalgia	16 results	“…a complicated medical history that included rheumatoid arthritis, fibromyalgia, diabetes mellitus” [35]		‒
7.	Cephal(al)gia	6 results	“…a superficial temporal artery biopsy for presumptive giant cell arteritis-induced *cephalalgia*” [35]		**Headache** (786 results in *JMCRs*)
8.	Mastalgia	1 result	“She could not recall any trauma to her chest and denied *mastalgia* or discharge from nipple” [35]		**Breast pain** (9 results in *JMCRs*)
9.	Arachnodactyly	4 results	“She had neither *arachnodactyly* nor thromboembolic events” [35]	**Arachne** was a master weaver and embroiderer. Bragging about her skill, Arachne challenged Pallas Athena to the competition, for which she was punished. The goddess, angered by the fact that Arachne wove the love affairs of Zeus, Poseidon, and Dionysus, tore the beautiful fabric woven by Arachne, and struck her four times on the forehead. The unfortunate Arachne could not survive such shame and hanged herself, but Athena pulled her out of the noose and turned her into a spider [36].	** Spider fingers** (0 results in *JMCRs*)
10.	Arachnoid	36 results	“Intracranial subdural empyema is an infection that is contained within the space between the dura and *arachnoid mater*” [35]		‒
11.	Arachnoid mater	10 results			
12.	Atlas	23 results	“Cervical myelopathy caused by atlantoaxial instability in a patient with an os odontoideum and total aplasia of the posterior arch of the *atlas*: a case report” [35]	“In Greek mythology, **Atlas** was a Titan whose bulk surpassed that of any other man, and who was condemned to support the heavens on his shoulders” [37]	** First cervical vertebra** (2 results in *JMCRs***)**
13.	Atlanto-occipital joint	3 results	“Her *atlanto-axial* and *atlanto-occipital joints* demonstrated full range of motion” [35]		‒
14.	Atlanto-axial joint	4 results			‒
15.	Atropine	36 results	“He was given two doses of atropine and then ephedrine without an increase in heart rate” [35]	**Atropos** was the oldest of the three fates (Moiras) who spun the thread of human destiny. “Atropos was the one who cut the thread when it was time for someone to die;… Atropine occurs in Solanaceae plants, especially the deadly nightshade plant, which was used in the middle ages to produce obscure and prolonged poisoning. Therefore, Carl von Linné named it *Atropa belladonna* after Atropos who cuts the thread of life” [37].	**Daturin** (0 results in *JMCRs*)
16.	Caput medusae	5 results	“The abdominal wall appearance revealed a *caput medusae* due to portal hypertension” [35]	**Medusa** was a monster with a woman’s face and snakes instead of hair [36].	= **Palm tree sign** (0 results in *JMCRs*), **Medusa’s head** (0 results in *JMCRs*)
17.	Chimera	5 results	“…a study demonstrated that in mouse bone marrow *chimeras* with CD80/86 knockout B-cellsб resistance to the induction of proteoglycan-induced arthritis was present” [35]	**Chimera** was a mythical monster with the head of a lion, the body of a goat, and the tail of a snake, from whose mouth erupted flames. Chimera was killed by Bellerophon (Βελλεροφόντης), grandson of Sisyphus.	**‒**
18.	Coma	329 results	“Hypertriglyceridemia as a possible cause of *coma*: a case report” [35]	**Comus** was “the guardian of banquets and indulged in nightly orgies which resulted in a state of profound insensibility caused by a drunken stupor” [37]	‒
19.	Cyclopia	4 results	“*Cyclopia* with shoulder dystocia leading to an obstetric catastrophe: a case report” [35]	**Cyclopes** were “one-eyed, gigantic and lawless shepherds in Sicily who devoured human beings” [37]	**Synophthalmia** (2 results)
20.	Echocardiography	605 results	“*Cardiac echo* showed mild mitral and tricuspid regurgitations without abnormal aortic valve” [35]	**Echo ** was a nymph, who was punished by Hera, for distracting her with long conversations, while Zeus was cheating on her with other nymphs. As a result, Echo could only repeat the endings of phrases or words of others.	‒
21.	Echocardiogram	412 results			
22.	Cardiac echo	2 results			
23.	Echo	254 results			
24.	Echolalia	7 results	“She was only able to produce a word salad and showed *echolalia*” [35]		**‒**
25.	Ether	6 results	“Allergic contact dermatitis from 2,3-dibromocresylglycidyl ether has been reported” [35]	**Aether** is the personification of light who organized the cosmic matter in the sky [37].	‒
26.	Hebephrenia	1 result	“Kraepelin grouped together catatonia, *hebephrenia* and paranoid psychosis, as dementia praecox” [35]	**Hebe ** was the daughter of Zeus and Hera. She was the goddess of youth [37]. The term “hebephrenia” refers to the more prominent appearance of the disorder in patients around puberty.	**Disorganized schizophrenia** (0 results)
27.	Hemeralopia	1 result	“This report presents a case of a 28-year-old man consulting for a progressive fall of visual acuity with *hemeralopia*” [35]	**Hemera** was the Greek goddess of the day [36]	**Day blindness** (1 result)
28.	Hermaphroditism	8 results	“Testicular seminoma has rarely been reported in patients with true *hermaphroditism*” [35]	**Hermaphrodite ** was the son of Hermes and Aphrodite, a young man of unprecedented beauty who was loved by the nymph Salmakida. However, not achieving reciprocity, she turned to the gods with a request that they create one creature from them—a half-man-half-woman.	**Ovotesticular disorder** (2 results)
29.	Hygiene	120 results	“Inclusion criteria were: good oral *hygiene*, absence of lesions of the oral mucosa, no smoking” [35]	**Hygieia** is the goddess of health, the eldest daughter of Asclepius, who was often depicted together with her father [36].	**‒**
30.	Hymen	4 results	“Hysteroscopy- and laparoscopy-based diagnosis and treatment of girls with unbroken *hymen* with an obstructing uterine septum: two case reports” [35]	**Hymen** is the god of marriage and the personification of the wedding feast [37].	**‒**
31.	Iris	79 results	“Pathology characteristics of ocular von Hippel-Lindau disease with neovascularization of the *iris* and cornea: a case report” [35]	**Iris** was the daughter of Taumantus and Electra, the goddess of the rainbow, who appears after rain in splashes of water or in a cloud, the messenger of the gods, and a mediator between gods and people [36].	**‒**
32.	Iridocyclitis	14 results	“…no slit-lamp examination was performed, and she had no indication of anterior uveitis or *iridocyclitis*” [35]		‒
33.	Iritis	15 results	“Her treating ophthalmologist diagnosed acute *iritis* with secondary glaucoma” [35]		**‒**
34.	Cofactor Klotho	3 results	“Loss of function in PHEX is associated with increased circulating FGF23 which acts to reduce expression of sodium-phosphate co-transporters (NaPi) in the renal tubule in association with its *co-factor Klotho*, and to reduce 1α-hydroxylase activity” [35]	**Klotho** is one of the three Moiras, or Fates, who spins the thread of human life [37].	–
35.	Klotho gene	1 result	“…the prolonged routine consumption of thousands of international units of vitamin D may interfere with the regulation of phosphate homeostasis by fibroblast growth factor-23 and the *Klotho gene* product” [35]		
36.	Klotho protein	1 result	“A markedly decreased expression of *Klotho* protein in a hyperplastic parathyroid gland is present in patients on HD” [35]		
37.	Labyrinth	8 results	“The pathogenesis of serous *labyrinthitis* in our patient may be due to toxins present in the *labyrinth*” [35]	**Labyrinthus** was an amazing maze, built near Knossos by the skilled Athenian craftsman Daedalus by order of the Cretan King Minos. The Labyrinth consisted of thousands of different rooms, and the Minotaur was hidden there [36].	** = Otic capsule** (5 results)
38.	Labyrinthitis	3 results			**‒**
39.	Lethargy	134 results	“A 67-year-old Japanese man presented to our hospital with generalized weakness, *lethargy*, and weight loss” [35]	**Lethe** is one of the six rivers of the underworld. According to the beliefs of the ancient Greeks, the dead, having entered the kingdom of Hades, drank water from this river and forgot everything they saw and experienced in this life to be reincarnated in another life [36]	**‒**
40.	Morphine	83 results	“All included patients previously underwent therapy with *morphine* and NSAIDs previously but were resistant to treatment” [35]	**Morpheus** is the Greek god of dreams, and the son of the god of sleep, Somnus [37]	–
41.	Narcissistic personality disorder	1 result	“He was also diagnosed with co-morbid *narcissistic personality disorder*” [35]	**Narcissus** was “the son of the river god Cephissus, and the embodiment of self-conceit. He fell in love with his own image reflected in the water of a river, to the extent of trying to talk to it and embrace it. He finally died of longing for his own image. Narcissism as a psychological term was coined in the late 1890s to describe a specific sexual perversion” [37].	**‒**
42.	Narcissistic personality traits	1 result	“…poor coping may be part of a personality disorder, such as borderline personality or dependent and *narcissistic personality traits*” [35]		**‒**
43.	Nycturia	1 result	“Accessory symptoms are *nycturia*, headache, intellectual deterioration” [35]	**Nycta** is the Greek goddess of the night, the antipode and mother of the goddess of the day—Hemera, the daughter of Chaos [36].	–
44.	Panacea	1 result	“Hundreds of millions of people in polyparasitized poor communities around the world are taking ivermectin to combat various diseases, making it a *panacea* for resource-poor countries” [35]	**Panacea** (literally means: “healer who heals everything”) is one of the four daughters of the god of healing Asclepius, she is the patroness of medicinal treatment [36].	
45.	Panic	28 results	“This report describes a patient suffering from *panic* disorder who developed repeated suicidal ideation” [35]	**Pan** is the “ancient Greek god of woods, fields, shepherds, and flocks. He amused himself by giving lonely travelers sudden fright, and thus the origin of the eponym” [37].	–
46.	Proteus	27 results	“*Proteus mirabilis* is the second most common pathogen that causes urinary tract infections after *Escherichia coli*” [35]	**Proteus** is the son of Poseidon and Hera, a sea deity who can transform into various plants, animals (bull, boar, lion, monkey, panther), and birds, as well as take the form of natural elements (fire or water). In a figurative sense, Proteus means “changeable nature”, “inconstant person”, “cunning” [36].	–
47.	Psyche	2 results	“…the influence of the skin on the *psyche* (*somatopsychic* pathway), which could be implicated in this disease” [35]	**Psyche** is the embodiment of the soul and breath. In ancient Greek, the word “ψυχή” has several meanings: (1) breath, soul, and consciousness, (2) life, (3) mental qualities and character, (4) mood and feelings, and (5) essence, personality, and person [36].	–
48.	Somatopsychic	1 result			–
49.	Psycho-syndrome	2 results	“After extubation, the patient presented a mild *psycho-syndrome* with cognitive slowing and deficits in mnestic function” [35]		–
50.	Psychobehavioral	1 result	“Medical, neurobiological, and *psychobehavioral* perspectives of mastocytosis: a case report” [35]		–
51.	Psycho-affective	1 result	“Given its *psycho-affective* properties of inducing euphoria, disinhibition and sexual arousal, the drug was later used as an adjunct to psychotherapy” [35]		–
52.	Neuropsychological symptoms	4 results	“A 76-year-old Caucasian woman presented with progressive left-sided hemiparesis, accompanied by hypoesthesia, hypoalgesia and *neuropsychological* symptoms” [35]		–
53.	Psycho-neuro-immunomodulatory	1 result	“Our study underlines the influence of the psyche on mast cell degranulation (psycho-neuro-immunomodulatory pathway)” [35]		–
54.	Antipsychotic drugs/medications	57 results	“Anti-inflammatory effects of antidepressant and atypical *antipsychotic* medication for the treatment of major depression and comorbid arthritis: a case report” [35]		–
55.	Antipsychotics	81 results	“Diabetic control and atypical antipsychotics: a case report” [35]		–
56.	Psychosis	81 results	“Williams syndrome and *psychosis*: a case report” [35]		–
57.	Psychotic	85 results	“Acute manic state with psychotic features in a teenager with autoimmune encephalitis: a case report” [35]		
58.	Psychopathic	1 result	“However, the patient should be treated first at a *psychopathic* ward” [35]		–
59.	Psychotherapy	38 results	“Many medical treatments and *psychotherapy* techniques were proposed for detoxification” [35]		–
60.	Psychiatry	112 results	“This condition has been well described in the surgical literature, but less reported in *psychiatry*” [35]		–
61.	Psychiatric	277 results	“*Psychiatric* reaction of an intensive care unit survivor in the context of coronavirus disease 2019: a case report” [35]		
62.	Priapism	7 results	“Using cyproterone acetate to treat recurrent ischemic *priapism* in a patient with sickle cell anemia as a comorbidity: a case report” [35]	**Priapus** was the son of Dionysus and Aphrodite, the god of fertility, the guardian of vineyards, apiaries, gardens, and fields. Priapus was born with a small, ugly body and unusually large genitalia [36].	–
63.	Sirenomelia	6 results	“Sirenomelia in a Nigerian triplet: a case report” [35]	“A **siren** is a fabulous monster, part of woman, part bird, who lured sailors to their destruction by enchanting singing. Sirens were confused with mermaids in the English literature of the fourteenth century. Therefore, designation as mermaid deformity is more appropriate than sirenomelic deformity on the basis of both mythological sources and morphological structure” [37]	**Mermaid syndrome** (3 results)
64.	Sphincter	118 results	“Biliary type-II *sphincter* of Oddi dysfunction with a pancreatic duct dilation: a case report and review of the literature” [35]	**The Sphinx** is a “hybrid monster, usually described as having the head of a woman and the winged body of a lion. The Sphinx asked a riddle of all travelers who passed by. Those who could not provide the correct answer were squeezed to death by the embrace of the Sphinx, and thus the similarity to the contraction of sphincters” [37].	–
65.	Syringe	45 results	“Because careful aspiration with a 2 mL syringe did not show any blood or cerebrospinal fluid, 1.0 mL of contrast medium was injected” [35]	**Syrinx** was an “Arcadian nymph who was chased by Pan, the hoofed and horned god of woods and fields. As Pan embraced her, Syrinx changed into a tuft of reeds, and the air going through the reeds produced such a lovely melody that he made a musical instrument out of them” [37].	–

Bold marking highlights the sources of the eponymic terms and their alternatives, it serves for better visualization and just a position of the corresponding lexical phenomena

The group of eponyms rooted in Roman mythology ranks second, and these terms are much less prevalent in modern medical case reports (6 eponyms, 113 results). Table 2 presents eponyms from this group.

**Table 2:** Eponyms rooted in the Roman mythology

No.	Terms with onomastic components	Number of results in *JMCRs* as of 2022	Examples from *JMCRs*	Reference information	Alternative terms (if available)
1.	Cupid’s bow	1 result	“She held her mouth mostly opened with a *cupid bowed* upper lip, full lower lip, and a slightly protruding tongue” [35]	**Cupid** is the Roman god of love, desire, and affection, usually depicted with the archery bow [36].	–
2.	Janus kinase 2 (JAK2)	16 results	“The report discusses the differential diagnosis using erythropoietin, erythropoietin-receptor and *Janus kinase 2*” [35]	**Janus**: one of the most ancient Roman gods, depicted as a man with two faces on either side of his head [36].	–
3.	Semilunar incision	1 result	“a semi-lunar incision was made across the palatal aspect of at least 5 mm from the papillary crest” [35]	**Luna** is the divine personification of the Moon.	–
4.	Semilunar valve	1 result	“…all six semi-lunar valve regions of the aorta and pulmonary artery have the propensity to develop anlagen of the coronary arteries” [35]		–
5.	Mercury	29 results	“Cracked *mercury* dental amalgam as a possible cause of fever of unknown origin: a case report” [35]	**Mercury** is the son of the supreme god Jupiter and Maya. Mercury (in ancient Greek mythology—Hermes) is the winged messenger of the Olympian gods, the patron god of merchants and trade. In the Middle Ages, the term “mercurium” was applied to the chemical element of hydrargyrum, because of its mobility and fluidity [36].	**Hydrargyrum** (0 results in *JMCRs*)
6.	Venereal disease	65 results	“His cerebrospinal fluid examination showed a nonreactive *venereal* disease research laboratory test” [35]	**Venus** is the Roman goddess of beauty, love, and marriage [36].	= **Sexually transmitted disease** (29 results in *JMCRs*)

Bold marking highlights the sources of the eponymic terms and their alternatives, it serves for better visualization and just a position of the corresponding lexical phenomena

Four eponyms (88 results) stem from other world mythologies (Germanic and Egyptian). Table 3 demonstrates our findings in this group.

**Table 3:** Eponyms rooted in other world mythologies

No.	Terms with onomastic components	Number of results in *JMCRs* as of 2022	Examples from *JMCRs*	Reference information	Alternative terms (if available)
1.	Ammonia	66 results	“High serum levels of *ammonia* can cause neurotoxicity” [35]	The Egyptian god **Amun** is the equivalent of the Greek god Zeus in the perception of ancient people. The term is derived since ammonia was extracted near the temple of Ammon in Egypt [37].	–
2.	Hyperammonemia	20 results	“*Hyperammonemia* of unknown cause in a young postpartum woman: a case report” [35]		–
3.	Elf-like face	1 result	“…clinical findings include distinct facies (*elf-like face*), cardiovascular abnormalities, growth retardation	“In early Teutonic mythology, **elves** were supernatural beings of dwarfish form with magical powers and well-known for capricious interference in human affairs” [37]	**Elfin (elven) facies** (0 results)
4.	Ondine curse	1 result	“Classically, it presents as sudden death in infancy because of a failure of autonomic control of ventilation during sleep” (*Ondine curse*) [35]	In one of the versions of the German myth, **Ondine** was “a water nymph who was jilted by her husband for a mortal woman. For punishment, she took away his autonomic functions, including that of breathing, so that he had to consciously remember in order to breathe” [37].	**Central hypoventilation syndrome** (4 results)

Bold marking highlights the sources of the eponymic terms and their alternatives, it serves for better visualization and just a position of the corresponding lexical phenomena

Two terms with onomastic components come from the Bible (15 results). Table 4 present our results.

**Table 4:** Eponyms rooted in the Bible

No.	Terms with onomastic components	Number of results in *JMCRs* as of 2022	Examples from *JMCRs*	Reference information	Alternative terms (if available)
1.	Barbiturates	14 results	“GHB intoxication resembles intoxication with sedative-type drugs such as *barbiturates*” [35]	“In 1863, Johan Adolf Baeyer discovered malonylurea, a substance that would become the parent compound of the barbiturates. The date of this discovery is believed to have been 4 December, and on that same day, Baeyer visited a tavern frequented by artillery officers. 4 December is the feast day of **Saint Barbara**, patron saint of artillery-men, as well as tunnellers and firemen; thus, he designated his malonylurea barbituric acid” [38]	–
2.	Lazarus phenomenon (Lazarus syndrome)	1 result	“Befittingly named the ‘*Lazarus phenomenon*,’ the recovery of spontaneous circulation after cessation of cardiopulmonary resuscitation is an extremely rare occurrence that was first described in 1982 and has been mentioned only 38 times in the medical literature” [35]	**“Lazarus** of Bethany died after an unspecified illness. He was raised from the dead after 4 days by Jesus” [37]	**Autoresuscitation** (1 result)

Bold marking highlights the sources of the eponymic terms and their alternatives, it serves for better visualization and just a position of the corresponding lexical phenomena

Within the corpus of analyzed MCRs, four eponyms stem from world literature (146 results) and are presented in Table 5.

**Table 5:** Eponyms rooted in world literature

No.	Terms with onomastic components	Number of results in *JMCRs* as of 2022	Examples from *JMCRs*	Reference information	Alternative terms (if available)
1.	Rapunzel syndrome	2 results	“Surgical outcome of jejunum-jejunum intussusception secondary to *Rapunzel syndrome*: a case report” [35]	**Rapunzel** is the long-haired girl from the fairy tale by the Brothers Grimm. The psychiatric term “Rapunzel syndrome” means intestinal obstruction due to the pathological desire of patients with some mental disorders to swallow their hair, which causes trichobezoars to form in the intestines [37].	–
2.	Pickwickian syndrome	1 result	“Based on the Charles Dickens’ character Joe, the fat boy in ‘The Posthumous papers of the Pickwick Club,’ Osler and later Burwell applied the name ‘*Pickwickian Syndrome*’ to the combination of obesity, hypersomnolence, and the signs of chronic alveolar hypoventilation” [35]	“In 1906, William Osler called obese, sleepy people **pickwickians** in reference to such a character in Dickens’s novel *The Pickwick Club*” [37]	**Obesity hypoventilation syndrome** (3 results)
3.	Munchausen syndrome	2 results	“*Munchausen syndrome* is a factitious disorder that involves falsification of psychological or physical signs or symptoms caused entirely by the patient themselves, in a clear state of consciousness, in order to play the role of a sick person” [35]	The eponym is derived from *The Adventures of Baron Munchausen* by the German writer Rudolf Erich Raspe. The protagonist—**Baron Munchausen**—constantly tells unbelievable, exaggerated, and dubious stories about his military exploits [37].	**Factitious disorder imposed on self** (0 results)
4.	Syphilis	141 results	“Congenital *syphilis*, still a reality in twenty-first century: a case report” [35]	“Syphilis was named after the poem *Poetical History of the French Disease*, written by Hieronymus Fracastorius (Giorolama Fracastoro) in 1525. The hero of the poem, **Syphilus**, was a shepherd whose flock was dying in the parched land from extreme heat and thirst. He cried out against the Sun and persuaded others to no longer worship him” [37]. As a result, the Sun punished Syphilus, who is supposed to be the first sufferer from this disease.	**Lues** (4 results), **Lues venerea** (0 results)

Bold marking highlights the sources of the eponymic terms and their alternatives, it serves for better visualization and just a position of the corresponding lexical phenomena

The research demonstrated that most medical eponyms in modern MCRs stem from Greek mythology. Table 6 demonstrates the etymological and quantitative distribution of terms with onomastic components in *JMCRs*.

**Table 6:** Etymological and quantitative distribution of eponyms in *Journal of Medical Case Reports*

Source	Number of eponyms	Number of results
Greek mythology	65	3633
Roman mythology	6	113
Other mythologies	4	88
The Bible	2	15
World literature	4	146
Total	81	3995

The results demonstrate that the majority of eponyms in *JMCRs* are rooted in Greek mythology (65 terms, 3633 results). The detected Greek eponyms represent a very extensive pantheon of Greek gods and heroes of primary and secondary importance. The group of eponyms rooted in Roman mythology ranks second, and these terms are much less prevalent in modern medical case reports (6 eponyms, 113 results). Four eponyms (88 results) represent other world mythologies (Germanic and Egyptian). Two terms with onomastic components come from the Bible (15 results), and four eponyms stem from modern literature (146 results).

Tables 7 and 8 demonstrate the etymological, thematic, and structural distribution of eponyms in *JMCRs.*

**Table 7:** Etymological and thematic distribution of eponyms in *Journal of Medical Case Reports*

Semantics	Source				
	Greek mythology	Roman mythology	Other mythologies	The Bible	World literature
Pathological conditions	32	1	3	1	4
Anatomical descriptions	10	2	‒	‒	‒
Medical instruments, diagnostic tools, and doctor’s manipulations	10	1	‒	‒	‒
Physiological phenomena	8	1	‒	‒	‒
Chemical substances and medications	5	1	1	1	‒

**Table 8:** Etymological and structural distribution of eponyms in *Journal of Medical Case Reports*

Structure	Source				
	Greek mythology	Roman mythology	Other mythologies	The Bible	World literature
One-word terms	48	1	2	1	1
Two-word phrases	11	5	1	1	3
Three-word phrases	6	‒	1	‒	‒

The research revealed that medical eponyms are most frequently used to refer to pathological conditions (41 eponyms). The majority of these eponyms are one-word terms (25), rooted in Greek mythology (arthralgia, myalgia, neuralgia, fibromyalgia, cephal(al)gia, mastalgia, arachnodactyly, coma, cyclopia, echolalia, hebephrenia, hemeralopia, hermaphroditism, iridocyclitis, iritis, lethargy, labyrinthitis, nycturia, panic, proteus, psychosis, psychotic, psychopathic, priapism, and sirenomelia), 1 eponym from the Egyptian mythology (hyperammonemia), and 1 more term from Italian literature (syphilis). There are also ten two-word phrases, four of which stem from Greek mythology (caput medusa, psycho-syndrome, psycho-affective, and neuropsychological symptoms), three more from the world literature (Munchausen syndrome, Pickwickian syndrome, and Rapunzel syndrome), and one each from Roman mythology (venereal disease), Germanic mythology (Ondine curse), and the Bible (Lazarus phenomenon). This thematic category also embraces 3 three-word phrases derived from the Greek (psycho-neuro-immunomodulatory, narcissistic personality disorder, and narcissistic personality traits) and Teutonic (elf-like face) mythologies. Our study confirmed that the terminology for pathological conditions is deeply rooted in the ancient Greek tradition [21], hence the considerable number of eponyms in this group.

The second most prevalent (12 terms) thematic group of medical eponyms is the category of anatomical descriptions. Among them, Greek eponyms also prevail: six one-word terms (arachnoid, atlas, hymen, iris, labyrinth, sphincter), two two-word phrases (Achilles tendon and arachnoid mater), two three-word phrases (atlanto-occipital joint and atlanto-axial joint). There are also two two-word phrases derived from Roman mythology (Cupid’s bow and semilunar valve).

The third most prevalent group contains 11 eponyms denoting medical instruments, diagnostic tools, and doctors’ manipulations. It embraces mostly one-word Greek terms (nine eponyms): echocardiography, echocardiogram, echo, hygiene, panacea, psychotherapy, psychiatry, psychiatric, and syringe, and one two-word Greek phrase: cardiac echo. There is also one two-word phrase, derived from Roman mythology (semilunar incision).

The thematic group of physiological phenomena embraces nine eponyms, derived from Greek mythology, including four one-word terms (chimera, psyche, somatopsychic, and psychobehavioral), three two-word phrases (cofactor klotho, klotho gene, and klotho protein), one three-word phrase (Achilles tendon reflex), as well as one two-word phrase stemming from Roman mythology (Janus kinase 2).

The group of chemical substances and medications is represented by eight one-word terms, stemming from Greek mythology (antipsychotics, atropine, ether, and morphine), Roman mythology (mercury), Egyptian mythology (ammonia), and the Bible (barbiturates), as well as one two-word phrase from Greek mythology (antipsychotic drugs).

Table 8 demonstrates the structural distribution of eponyms in *JMCRs*: one-word terms, two-word phrases, and three-word phrases.

The majority of one-word terms (48) account for Greek mythology. Our study demonstrated considerable word-building potential of the Greek language since this etymological source is the most productive in terms of term formation, for example, “-algia” (6 terms), “echo-” (5 terms), “psyche-” (15 terms), and so on. Two-word phrases are mostly represented by the construction “eponym + syndrome/disease/phenomenon, and so on”. Three-word phrases are not numerous, but contain important details about the terms via the additional lexemes.

## Discussion

Our findings contribute to the understanding of the prevalence and distribution of mythological and literary eponyms in modern medical literature. By focusing on the etymology of these eponyms in MCRs, our study provides a reference for medical professionals who may encounter and use these terms in their practice and research. The awareness of the origins of medical terms with onomastic components and their alternative terms, if available, will improve communication and understanding within the medical community. Overall, our research demonstrates that a significant number of eponyms in medicine are derived from sources beyond the medical field, indicating the cultural influence on the medical profession.

One can easily observe the paramount significance of the Greek language and cultural heritage in the contemporary English sublanguage of medicine. This is due to the rich informational and metaphorical arsenal of this ancient corpus of human knowledge. It is necessary to bear in mind that “virtually all genres of medical discourse are largely based on Latin and latinized Greek terminology” [21]. Hence, our study highlights the influence of classical Greek mythology on medical language and its importance in describing medical conditions and structures. By understanding the etymology and thematic distribution of eponyms, healthcare professionals can better appreciate the cultural and historical significance of medical language.

As for Roman mythology, the analysis of Table 2 revealed a total of six eponyms with onomastic components derived from this source, namely “Cupid’s bow,” “Janus kinase 2” (JAK2), “semilunar incision,” “semilunar valve,” “Mercury,” and “venereal disease”. In the case of eponyms rooted in other world mythologies, our analysis shows that these eponyms are relatively rare in *JMCRs*, with only four terms identified. However, they offer an interesting insight into the influence of mythology on medical language. For example, the term “elf-like face” is derived from early Teutonic mythology, where elves were supernatural beings of dwarfish form with magical powers, well known for their capricious interference in human affairs. Similarly, the term “Ondine curse” is based on a German myth where a water nymph takes away the autonomic functions, including breathing, from her mortal husband as punishment. Among the eponyms derived from other world mythologies, the term “ammonia” was found to have the highest number of results (66). The term is derived from the name of the Egyptian god Amun (Ammon), who was the equivalent of Zeus in the perception of ancient Greeks. Eponyms rooted in the Bible are often used to describe medical conditions or treatments that have a connection to a biblical story. Eponyms rooted in world literature are based on the features of fictional characters, and they offer an interesting insight into the cultural influence on medical language. Future studies could expand the scope of the analysis to other biomedical journals and other databases to provide a more comprehensive overview of the use of eponyms rooted in Roman and other world mythologies, as well as those rooted in modern literature.

Our research sheds light on the types of medical concepts that are most commonly expressed using eponyms. In terms of thematic distribution, medical eponyms are most frequently used to refer to pathological conditions, and anatomical descriptions constitute the second most eponymized category, followed by medical instruments, diagnostic tools, doctors’ manipulations, physiological phenomena, and chemical substances and medications. Greek mythology is the primary source of eponyms in each category. Hence, our findings demonstrate that Greek mythology is the most frequently used source of eponyms in modern medical case reports, reflecting the continued relevance of ancient Greek culture in the field of medicine.

As for the structural peculiarities, the majority of eponyms are one-word terms, followed by two-word phrases. This tendency indicates a strong word-building potential of medical terminology, which is designed to be concise and precise, and one-word eponyms are often more effective in achieving these goals than longer phrases. Hence, the use of one-word terms allows for more laconic narration, which is essential in medical writing.

The study also identified alternative terms for some of the terms with onomastic components. For example, the term “achilles tendon” has an alternative term of “calcaneal tendon,” whereas the term “achilles tendon reflex” has alternative terms of “ankle-jerk reflex” and “ankle jerk reflex”. There are several terms with onomastic components that have multiple alternative terms. For instance, “myalgia” has 159 results and is synonymous with “muscle ache” (10 results in *JMCRs*), “muscle pain” (40 results), and “muscular pain” (5 results). In contrast, some terms with onomastic components do not have alternative terms, such as “fibromyalgia”, “panacea”, and “lethargy”.

As to the strategy of using either an eponym or an equivalent descriptive term (if available), most examined MCRs tend to use terms with onomastic components (see Tables 1–5). For instance, we found 65 results for “venereal disease” versus 29 results for “sexually transmitted disease,” 5 results for “caput medusae” versus 0 results for “palm tree sign,” 42 results for “Achilles tendon” versus 2 results for “calcaneal tendon,” and so on. An undeniable benefit of Greek-based eponyms is the fact that these terms are internationally used and immediately understood worldwide. Moreover, the Greek terms ensure the brevity and conciseness of terms as contrasted to lengthy descriptive terminological collocations, and this feature is of paramount importance for medical writing in general and MCRs in particular [34].

However, there are few exceptions to this tendency, as with the term “headache” (786 results in *JMCRs*), which obviously prevails over the eponymic “ceph(a)algia” (6 results). Similarly, “arthralgia” has 154 results, and is synonymous with “joint pain” (629 results in *JMCRs*), and “painful joints” (5 results). Another exception is the eponym “Ondine curse” (1 result) as opposed to “central hypoventilation syndrome (4 results). In this context, the scholars observe that “there has been some negative reaction to “Ondine’s curse” as a diagnostic term. One objection is that there are other versions of the Ondine myth in which she does not afflict her husband with a curse. A somewhat more cogent objection is that Ondine’s husband exhibited other effects of loss of autonomic function, such as disorders of his five senses and muscles, unlike patients with this syndrome” [37]. Thus, the knowledge of prevailing tendencies and existing debatable issues in the medical community is essential when using an eponym or its equivalent.

In the process of our search, we encountered 22 cases of “erroneous” medical eponyms, which required exclusion from the sampling. One reason for this exclusion was the authors’ mentioning proper names, fully coinciding in graphic form with some mythological denotations, for example, surnames, and the names of laboratories or devices. We found ten cases of an “erroneous” Biblical eponym with the name “Lazarus” (five articles) [39–43], for example, “CDH was first described in 1679 by *Lazarus* Riverius” [39], “They were first described by *Lazarus* and Trombetta in 1978” [42], and “*Lazarus* and Chiang examined patients that had a bone marrow transplant later followed by a solid organ” [43]. There were four cases of “pseudo” Roman eponyms with the name “Hercules” (four articles) [44–47], for example, “After incubation at room temperature for ten minutes, the absorbance was measured at 540 nm in a spectrophotometer (Bio-Rad Laboratories, *Hercules*, CA, USA)” [45], and one case for “Venus”: “We report the unusual case of a girl diagnosed with *Neisseria gonorrhoeae* after bathing in a heavily frequented hot pool at the edge of the crater lake Specchio di Venere (“Mirror of *Venus*”) on Pantelleria Island, Italy” [48]. As a result, we did not include the eponym “the Hercules complex” from Roman mythology [36] in our study. We also found one erroneous case for “Sphinx”: “*Sphinx* YAG:holmium laser stone fragmentation” [49]. Hence, the eponym “face of Sphinx” from Egyptian mythology [36] was also excluded. Another reason could be a misprint, which also distorted the search results, for example, in the term “venous”: “In cases such as a twin pregnancy, the gravid uterus increases intra-abdominal pressure significantly and decreases the epidural and subarachnoid space by the associated engorgement of the epidural venus plexus” [50]. We also detected several mistakes in the spelling of some eponyms, which can be confused with mythological or literary terms, for example, in “Adam’s forward bend testing”—three cases in two papers [51, 52] and “Adam’s solution”—two cases in one paper [53], which should be spelled “Adams’ forward bend testing” and “Adams’ solution,” as these medical phenomena were named after Dr. Adams, not Adam the first human from the Bible. It is our opinion that the awareness of an eponym’s etymology can effectively prevent and minimize the appearance of such errors in medical case reports.

Our study demonstrated that mythological and literary eponyms are ever-present and deeply incorporated into the modern medical discourse, which necessitates a considerable amount of encyclopedic knowledge. The adequate use of eponyms in medical case reports and understanding of their etymology are essential prerequisites for doctors’ all-round development, and successful communication of one’s clinical findings to international colleagues in research. Using these terms promotes the laconic manner, brevity, and conciseness of medical case reports, since eponyms are internationally adopted and understood. Moreover, the prevalence of mythological and literary eponyms in modern MCRs demonstrates that these terms are endowed with a powerful associative and imaginative potential—that is, they immediately express the nature of the disease, and so on, and contribute to better memorization due to their saturated metaphorical shades.

The findings contribute to our understanding of the etymology and distribution of eponyms in medical case reports and provide insights into the historical and cultural roots of medical terminology. The use of these terms contributes to better expressiveness in describing diseases and medical phenomena. At the same time, the appropriate use of eponyms requires a strong understanding of their etymology and prevailing tendencies. Medical professionals must strive to use eponyms and their equivalents in a responsible manner, recognizing the power of language in shaping medical knowledge and discourse. Therefore, we emphasize the need for medical students and practitioners to be proficient in Greek and Latin terminology to comprehend medical eponyms and better communicate with patients and colleagues. The continued relevance of ancient Greek and Latin in contemporary medical practice justifies the importance of incorporating the study of these languages in medical education.

This study highlights the need for a more interdisciplinary approach to medical education and the importance of the intersection between medicine and humanities. In particular, the results of this research will help in improving the writing proficiency among undergraduates and PhD students. Tables 1–5 present our findings in an accessible and comprehensive way, and therefore can be used in the teaching practice at a medical university for undergraduates and PhD students as part of a formal curriculum, as well as for healthcare specialists duping their continuing professional education. By providing noneponymous equivalents (if available), we not only display general tendencies in medical terminology and detect the prevalence of eponyms versus their descriptive alternatives, but also expand the terminological competence and professional vocabulary of medical students. We hope that this study will contribute to the ongoing discussion on the role of eponyms in medical writing, and encourages future research in this area. Further research is needed to explore the role of eponyms in medical communication and education.

## Conclusions

Our study provided a comprehensive overview of the use of mythological and literary eponyms in medical case reports and highlighted their importance in modern medical discourse. The use of eponyms facilitates communication, and the transfer of medical knowledge across linguistic and cultural barriers, ultimately contributing to the global advancement of medical science. The use of eponyms promotes brevity and laconism in medical case reports, and these terms have powerful associative and imaginative potential that contributes to better memorization. Our findings confirm the significant influence of the Greek language and culture on contemporary English medical sublanguage, reflecting the rich informational and metaphorical arsenal of this ancient corpus of human knowledge.

The research emphasizes the importance of understanding the etymology and origins of medical terminology, as it can provide valuable insights into the historical and cultural context of medical practice. In this context, it is crucial for medical professionals to have adequate knowledge of these terms to facilitate effective communication and understanding of clinical findings. Therefore, it is essential to educate medical students on the most prevalent eponyms used in contemporary medicine and their origins to ensure their proper use. Medical students should not only focus on the technical aspects of their profession, but also develop a broader perspective that encompasses the humanities. We recommend that medical schools and institutions include the study of eponyms in their curricula to help medical students become familiar with these terms. This will enable them to appreciate the cultural and historical context of medicine and use eponyms appropriately, ensuring that medical knowledge is effectively communicated across linguistic and cultural barriers. We believe that incorporating the study of eponyms into medical education and continuing professional development will promote a deeper understanding of the field and contribute to the advancement of medicine. This approach will develop well-rounded healthcare professionals, who possess both professional expertise and broad background knowledge.

Overall, the integration of the humanities into medical education is crucial for the holistic development of healthcare professionals and the advancement of medical science. Future studies could explore the use of eponyms in other areas of medical literature and across different languages and cultural contexts, expanding the scope of the analysis to include other databases, to provide a more comprehensive overview of the use of medical eponyms rooted in world mythology and literature.

## Data Availability

All quoted material and data are available in open access publications of *Journal of Medical Case Reports*.

## Abbreviations

*JMCRs*: *Journal of Medical Case Reports*
MCR: Medical case report
